# Hyperparasitism of *Acroclisoides sinicus* (Huang and Liao) (Hymenoptera: Pteromalidae) on Two Biological Control Agents of *Halyomorpha halys*

**DOI:** 10.3390/insects12070617

**Published:** 2021-07-08

**Authors:** Alberto Mele, Davide Scaccini, Alberto Pozzebon

**Affiliations:** Department of Agronomy, Food, Natural Resources, Animal and Environment, University of Padova, viale dell’Università 16, 35020 Legnaro, Italy; davide.scaccini@phd.unipd.it

**Keywords:** brown marmorated stink bug, biological control, hyperparasitism, *Trissolcus japonicus*, *Trissolcus mitsukurii*, *Acroclisoides sinicus*

## Abstract

**Simple Summary:**

The brown marmorated stink bug, *Halyomorpha halys* (Stål), is an invasive pest that causes losses to many crops in different parts of the world. Biological control programs using the egg parasitoids *Trissolcus japonicus* (Ashmead) and *Trissolcus mitsukurii* (Ashmead) have been proposed as sustainable strategies to control this pest. *Acroclisoides sinicus* (Huang and Liao) is another parasitoid that frequently emerged from *H. halys* egg masses, hypothesized to be a hyperparasitoid of *Trissolcus* spp. that can have a potentially negative effect on the outcome of biological control programs. Under laboratory conditions, we investigated the impact of *A. sinicus* on the two primary parasitoids of *H. halys*, assessing its host preference and exploitation capacity. *Acroclisoides sinicus* demonstrated a clear preference in parasitizing *T. mitsukurii*, while the impact on *T. japonicus* was relatively low. *Acroclisoides sinicus* can use volatiles emitted by egg masses to distinguish between those parasitized by *T. mitsukurii* from those parasitized by *T. japonicus* or unparasitized ones. The results obtained here suggest that the presence of the hyperparasitoid can affect the composition of *H. halys* parasitoid complex.

**Abstract:**

*Halyomorpha halys* (Stål) is an invasive Asian pest that causes severe crop losses on various crops. Nowadays, management strategies against this pest mainly rely on pesticide use, but biological control with egg parasitoids is considered the most promising long-term and sustainable solution. *Trissolcus japonicus* (Ashmead) and *Trissolcus mitsukurii* (Ashmead) are Asian egg parasitoids already present in Europe and are the most effective biological control agents of *H. halys*. Therefore, these two species are considered for biological control programs in Europe and other parts of the world. *Acroclisoides sinicus* (Huang and Liao) is a pteromalid parasitoid wasp that frequently emerged from *H. halys* egg masses collected in northern Italy. This species has been hypothesized to be a hyperparasitoid of *Trissolcus* spp. parasitoids. This study was carried out under laboratory conditions where *A. sinicus* was tested in no-choice and two-choice experiments to assess the host preference between *T. japonicus* and *T. mitsukurii*. Olfactory responses of *A. sinicus* from volatiles emitted from different potential hosts were also tested. In all trials, *A. sinicus* showed a clear preference for parasitizing *H. halys* eggs previously parasitized by *T. mitsukurii* compared to *T. japonicus.* In no-choice experiments, the impact of the hyperparasitoid on *T. japonicus* was low, showing an exploitation rate of 4.0%, while up to a 96.2% exploitation rate was observed on *T. mitsukurii*. *Acroclisoides sinicus* was also attracted by volatiles emitted by egg masses parasitized by *T. mitsukurii*, while no response was observed to egg masses parasitized by *T. japonicus* or not parasitized. Therefore, according to the results obtained here, *A. sinicus* could limit the population development of *T. mitsukurii,* while lesser effects are expected on *T. japonicus*.

## 1. Introduction

The brown marmorated stink bug, *Halyomorpha halys* (Stål) (Hemiptera: Pentatomidae), is an invasive Asian pest that can infest various crops causing economic losses in many countries [1]. In Italy, after its first detection [2], *H. halys* caused more than 50% of losses to fruit crops, but it also caused severe losses to other crops and ornamentals [3,4,5,6]. Chemical control based on broad-spectrum insecticides is the most widely used strategy. However, due to the low susceptibility to various insecticide residues and the high mobility and reproductive rate of *H. halys*, many insecticide applications are required for its control, making this approach environmentally and economically unsustainable [1]. Among alternatives to chemical control in *H. halys* management, biological control is currently considered the most promising strategy. Research has been devoted to investigating the natural enemy complexes in native and invaded areas [7,8,9,10,11]. Species of the genus *Trissolcus* (Hymenoptera: Scelionidae) are egg parasitoids that show promising results for *H. halys* control. In particular, *Trissolcus japonicus* (Ashmead) is considered the most effective natural enemy in Asia, and it has been used in biological control programs both in North America and Europe [7,12,13]. Another egg parasitoid, *Trissolcus mitsukurii* (Ashmead), has been recorded to commonly parasitize *H. halys* egg masses with high parasitism rates in Japan [14]. In Europe, *Anastatus bifasciatus* (Geoffroy) (Hymenoptera: Eupelmidae) is an egg parasitoid native to Europe that successfully exploits *H. halys* eggs; thus, it has been considered for augmentative biological control strategies, but its performance in field conditions is inconsistent [15,16,17]. *Trissolcus kozlovi* Rjachovskij (Hymenoptera: Scelionidae) is another European parasitoid that has been found to exploit *H. halys* egg masses in Italy [8,9] and considered for biological control programs [18].

Adventive populations of Asian parasitoids have been discovered outside their native areas. *Trissolcus japonicus* was found in North America in 2014 [19], and in Europe in Switzerland in 2017 [20], Italy in 2018 [21] and recently in Germany [22]. The earliest European record of *T. mitsukurii* occurred in Italy in 2016 [9], and it has spread to other regions of northern Italy [8,21]. Furthermore, a biological control program with inoculative releases of *T. japonicus* was initiated for the first time in Italy in 2020 [23].

Wasps of the genus *Acroclisoides* (Girault and Dodd) (Hymenoptera: Pteromalidae) are known as facultative or obligate hyperparasitoids, and *Acroclisoides sinicus* (Huang and Liao) (senior synonym of *A. solus* (Grissell and Smith) [24,25]) was found to emerge from pentatomid eggs previously parasitized by primary parasitoid species both in North America and Europe [8,24,25]. Information on the origin of *A. sinicus* is still lacking, and the current hypothesis based on genetic diversity and distribution of other *Acroclisoides* species suggests it was recently introduced in Europe and the USA, possibly following the same pathways of *Trissolcus* spp. [25]. Nevertheless, according to available literature, *A. sinicus* is associated with several pentatomid (Hemiptera: Pentatomidae) species on three continents: *Euschistus* sp., *Brochymena* sp., *Chinavia hilaris* (Say) and *H. halys* in North America; *Plautia stali* (Scott), *Erthesina fullo* (Thunberg) and *H. halys* in Asia; *Palomena prasina* L., *Arma custos* (Fabricius), *Nezara viridula* L. and *H. halys* in Europe [7,8,25]. Laboratory studies on *Acroclisoides* suggested that they can successfully exploit pentatomid eggs only if previously parasitized by a primary parasitoid [25,26,27]. Moreover, molecular analysis of meconia of *H. halys* parasitized eggs suggested that *A. sinicus* was associated with *Trissolcus* spp. [28]. Recent findings confirmed what was previously supposed, that *A. sinicus* acts as an obligate hyperparasitoid of pupae of Scelionidae but not of Eupelmidae [29].

Obligate hyperparasitoids occupy the highest level in multitrophic systems, and they can negatively influence the success of biological control strategies [30,31]. In northern Italy, *T. japonicus* and *T. mitsukurii* coexist in many of the areas where they are distributed [7,32]. In the same areas, *A. sinicus* was often found in both cultivated and semi-natural areas [8,9,25]. Furthermore, *A. sinicus* preference among scelionid parasitoid species is still unexplored. In this study, host specificity of *A. sinicus* was tested through no-choice and two-choice tests. The olfactory response of *A. sinicus* to volatile cues from different host sources was tested in olfactometer experiments. We performed insect dissections to assess egg loads at different times after adult emergence.

## 2. Materials and Methods

### 2.1. Origin of Insects

In the experiments, we used egg masses of *H. halys* reared in laboratory colonies established in 2020. Stink bugs were reared in insect cages (30 × 30 × 30 cm; BugDorm-1, MegaView Science Co., Ltd., Taiwan, China) at 26 ± 5 °C, 60 ± 5% RH and 16:8 (L:D) photoperiod and fed with fresh vegetables (green beans and carrots), fruits (apples and kiwifruit) and sunflower seeds. Water was provided ad libitum with water-soaked cotton balls. Within the rearing cages, paper towels were provided to stink bugs as an oviposition substrate. Twice a week, food and water were replaced, and egg masses were collected from paper towels or, less commonly, from fruits or cage walls. After collection, egg masses were preserved in a climatic chamber at 7 °C for no more than 2 weeks before being employed in this study. *Trissolcus japonicus* and *T. mitsukurii* were obtained from *H. halys* egg masses collected in the field in different sites in northeastern Italy since 2019. *Trissolcus* species were morphologically identified using a stereomicroscope (Stemi 508, Carl Zeiss Microscopy GmbH, Jena, Germany) following the key by Talamas et al. [33]. Both *Trissolcus* colonies were maintained on *H. halys* egg masses and reared at 26 ± 2 °C, 60 ± 10% RH, and 16:8 (L:D) photoperiod; honey was provided as food.

In late August 2020, an *A. sinicus* colony was established starting from individuals obtained from field-collected egg masses that appeared to be parasitized by *T. mitsukurii* according to the characteristics of the egg’s meconia [34]. Identification of *A. sinicus* was performed using a stereomicroscope (Stemi 508, Carl Zeiss Microscopy GmbH, Jena, Germany) following the description by Grissell and Smith [24] and remarks in Sabbatini-Peverieri et al. [25]. The *A. sinicus* colony was maintained on *T. mitsukurii*-parasitized *H. halys* egg masses. Hyperparasitoids were fed with honey and they were used for no-choice and two-choice experiments after 12–14 days. All trials were conducted in a climatic chamber at 26 ± 2 °C, 60 ± 10% RH and 16:8 (L:D) photoperiod and honey was provided as food. All *A. sinicus* females used in the experiments were without previous oviposition experience (i.e., naïve) and, before the experiments, were allowed to mate in colonies for at least one day since their emergence.

### 2.2. No-Choice Tests

In the first experiment, we conducted a no-choice test by exposing naïve *A. sinicus* females to three types of *H. halys* egg masses: unparasitized, previously parasitized either by *T. japonicus* or by *T. mitsukurii*. The experimental unit consisted of a 50 mL Falcon tube with a hole covered by an insect-proof net for ventilation. In one experimental unit, a single 14-day-old mated female of *A. sinicus* was exposed to one *H. halys* egg mass. Parasitized egg masses were used after 4 days from the exposure to primary parasitoids. Parasitized and unparasitized egg masses used in the trial were all of the same age (i.e., collected the same day from *H. halys* rearing). Hyperparasitoid females were kept with the egg mass parasitized by *T. japonicus* or parasitized by *T. mitsukurii*, left ovipositing for 1 day or 3 days, and then removed. Each treatment was replicated twenty times. Honey was provided as food.

In a second no-choice test, a single-mated 14-day-old *A. sinicus* female was placed in an experimental unit described above, with an egg mass parasitized by *T. japonicus* or *T. mitsukurii* and left ovipositing for 1 day and then removed. Egg masses were all of the same age (i.e., collected the same day from *H. halys* rearing). Hyperparasitoid females were placed in the experimental unit after 2, 3, 4, 5, 6, or 7 days from the *Trissolcus* oviposition. Ten replicates were performed for each day and primary parasitoid species. Further, ten parasitized egg masses for each parasitoid species were not exposed to *A. sinicus* to assess the natural mortality of primary parasitoids.

Hatched and unhatched *H. halys* eggs were counted at the end of the experiment. All unhatched eggs were dissected under a stereomicroscope (Stemi 508, Carl Zeiss Microscopy GmbH, Jena, Germany), and the number of aborted *H. halys* eggs, aborted parasitoids and aborted hyperparasitoids were counted. All parasitoids that emerged from egg masses were counted and sexed; adult dissection for determining the presence of the ovipositor or ovaries helped in sex discrimination.

### 2.3. Two-Choice Test

A single-mated 14-day-old *A. sinicus* female was placed in a small arena (90 mm Petri dish) with two *H. halys* egg masses with a standardized number of eggs (25 eggs), one previously parasitized by *T. japonicus* and the other by *T. mitsukurii*; egg masses were both parasitized 4 days prior. One-hour direct observation of the arenas was carried out in a room with scattered light (to avoid biased choices due to light direction) at 26 ± 5 °C and 40 ± 10% RH; the choice was recorded when *A. sinicus* probed the egg mass for more than 1 min. After the 1 h observation, the female of *A. sinicus* was left on the arena, which was then transferred to the climatic chamber. After 24 h, each *A. sinicus* female was removed from the arena. For this trial, we performed 40 replicates, each one with a different *A. sinicus* female.

Finally, emerged parasitoids were sexed and counted, and the number of aborted eggs, aborted parasitoids, and aborted hyperparasitoids were recorded as described for the no-choice tests.

### 2.4. Olfactometer Trial

The olfactory response of *A. sinicus* to volatile cues emitted by parasitized and unparasitized egg masses was tested in the laboratory using a Y-tube glass olfactometer (stem: 100 mm; arms: 100 mm at a 30° angle between arms; internal diameter: 20 mm; outside diameter: 22 mm). According to some previously published literature, we mounted the olfactometer on a 40° sloped plane, since insects did not respond to volatile cues if the olfactometer was placed horizontally [35,36]. The central stem was attached to a vacuum pump that creates an airflow of 0.5 L/min throughout the olfactometer. The Y arms were connected to two glass sample chambers in which egg masses were placed. By two silicon tubes, glass chambers were connected to a glass jar filled with water that bubbles with airflow movement. Before the glass jar, a charcoal filter was placed to filter the air in the entrance. A cardboard box surrounded the olfactometer to minimize lights from the room; diffused light was provided by reflecting two LED lamps on a white screen placed at the beginning of the Y arms. The temperature in the black box was maintained at 26 ± 1 °C during the entire experiment.

During the experiment, a single egg mass with 25 eggs was placed in the glass chambers. In the experiment, we used egg masses after 5 days from the exposure to primary parasitoids and same-age unparasitized egg masses. Three different pairwise comparisons were performed: (i) egg mass parasitized by *T. japonicus* versus an unparasitized egg mass; (ii) egg mass parasitized by *T. mitsukurii* versus an unparasitized egg mass; (iii) egg mass parasitized by *T. mitsukurii* versus egg mass parasitized by *T. japonicus*. A single mated *A. sinicus* female was introduced in the central stem and observed for a maximum of 20 min. The choice was confirmed when *A. sinicus* entered in one or in the other Y arm for at least 2 cm from the junction of the arms. Each comparison was replicated 60 times. After each run, we switched the position of the two chambers, and every 10 runs, we cleaned the entire system using alcohol (96%), then acetone, and then rinsed it with tap water and let it dry.

### 2.5. Assessment of Acroclisoides sinicus Egg Load

The egg load in *A. sinicus* ovaries was carried out through dissection of females a few hours after emergence and after 1, 2, 4, 6, 10, 16, and 20 days from emergence from *T. mitsukurii* egg masses. *Acroclisoides sinicus* that emerged from one egg mass were kept together to ensure mating and with the remains of the egg mass from which they came from, since traces of host presence may stimulate ovary development [37]. However, no new parasitized *H. halys* egg masses were provided to these individuals. According to the procedure described by Jervis et al. [38], insects were killed by freezing, and dissection was performed under a stereomicroscope (Stemi 508, Carl Zeiss Microscopy GmbH, Jena, Germany) by gluing insects (dorsal side up, ventral down) on microscope slides and carrying out dissection in a droplet of saline solution (9 g NaCl/L). Insects were then sexed, and dissected ovaries were observed under a microscope (RML5, Mikroskop Technik Rathenow GmbH, Germany) for egg load count. Observed oocytes were considered mature when located at the end of the ovariole or in the lateral oviduct [38,39].

### 2.6. Statistical Analysis

For the analysis of the data generated by no-choice and two-choice experiments, we considered the following dependent variables: (i) the sum of the number of emerged and aborted *A. sinicus* and aborted *Trissolcus* over the number of eggs parasitized by *Trissolcus* (hyperparasitoid exploitation efficiency); (ii) the number of *A. sinicus* emerged over the number of eggs parasitized by *Trissolcus* (hyperparasitism efficiency); (iii) the number of *Trissolcus* emerged over the number of parasitized eggs (*Trissolcus* emergence); (iv) aborted *Trissolcus* over the number of eggs parasitized by *Trissolcus* (*Trissolcus* mortality from causes other than apparent parasitism by *A. sinicus*) [40]. All variables were considered with binomially distributed errors. For the analysis of the data obtained in the first no-choice experiment, the two *Trissolcus* species (*T. japonicus* and *T. mitsukurii*) and the two oviposition periods (1 day and 3 days) and their interaction were considered as independent variables and tested using a χ^2^ test (α = 0.05). Data obtained in the second no-choice test were also analyzed with a logistic regression considering the abovementioned dependent variables in separate analyses. *Trissolcus* species, days from primary oviposition and their interaction were considered independent variables and assessed using a χ^2^ test (α = 0.05). In both cases, multiple comparisons among means were performed with Tukey–Kramer post hoc methods (α = 0.05). A constant of 0.5 was added to data with zero value to handle overdispersion. All of the above models were run using the GLIMMIX procedure of SAS (ver. 9.4) [41]. The Fisher’s exact test (α = 0.05) was used to compare the host preference in two-choice and olfactometer trials using the FREQ procedure of SAS (ver. 9.4) [41]. The relationship between age of *A. sinicus* female and number of egg load was described with a polynomial regression, using the REG procedure of SAS (ver. 9.4) [41]. Residual plots were visually assessed for normal distribution and homoscedasticity and untransformed data were used in the regression.

## 3. Results

### 3.1. No-Choice Experiment

In the first no-choice test, 140 egg masses and 3525 eggs were used. The total number of *A. sinicus* individuals that emerged from *Trissolcus*-parasitized egg masses numbered 607, and the mean sex ratio of offspring from *T. mitsukurii*-parasitized egg masses was 89:11 female:male, and 56:44 female:male from *T. japonicus*-parasitized egg masses. The development time from female oviposition to the emergence of new adults ranged between 11 and 12 days but, in one case, a female took 30 days to emerge. In this experiment, 593 *A. sinicus* individuals developed in eggs previously parasitized by *T. mitsukurii*; only 14 were able to emerge from eggs parasitized by *T. japonicus*. A maximum of four *A. sinicus* emerged from *T. japonicus*-parasitized egg masses, while they were 16 and 27 on *T. mitsukurii*-parasitized egg masses in the 1-day and 3-day oviposition trial, respectively. Five hundred and eight *H. halys* nymphs hatched from fresh (i.e., not parasitized by the primary parasitoid) egg masses exposed to *A. sinicus* and this parasitoid never emerged. These 40 egg masses were excluded from analysis since the dependent variables considered were all equal to zero.

**Figure 1:**
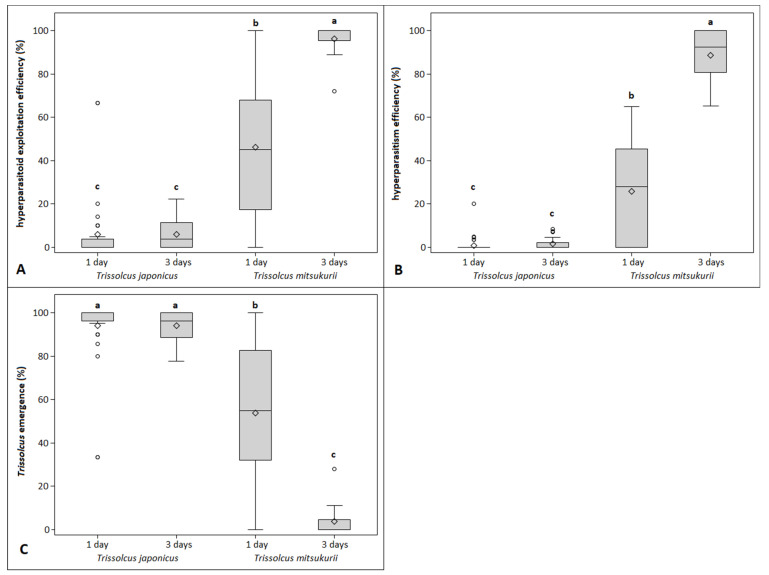
Boxplots showing hyperparasitoid exploitation efficiency (**A**), hyperparasitism efficiency (**B**) and *Trissolcus* emergence (**C**) observed during the first no-choice experiment for different *Trissolcus* species and *A. sinicus* oviposition periods. Different letters indicate significant differences at Tukey–Kramer test on the least-square means (α = 0.05). Bars represent minimum and maximum scores. Circles indicate outliers.

The hyperparasitoid exploitation efficiency was zero on non-parasitized *H. halys* egg masses, 4.0% on *T. japonicus*-parasitized egg masses and 63.9% on *T. mitsukurii*-parasitized egg masses. A higher performance of the hyperparasitoid was observed on *T. mitsukurii* as compared to *T. japonicus* (hyperparasitoid exploitation efficiency: χ^2^ = 1451.21, df = 1, *p* < 0.0001; hyperparasitism efficiency: χ^2^ = 1093.15, df = 1, *p* < 0.0001). Concerning the oviposition period, females of *A. sinicus* left for 3 days on the egg mass displayed higher performance than those that were left for just 1 day (hyperparasitoid exploitation efficiency: χ^2^ = 108.87, df = 1, *p* < 0.0001; hyperparasitism efficiency: χ^2^ = 46.85, df = 1, *p* < 0.0001). The interaction “host species*oviposition period” revealed significant differences for both variables (hyperparasitoid exploitation efficiency: χ^2^ = 55.61, df = 1, *p* < 0.0001; hyperparasitism efficiency: χ^2^ = 8.62, df = 1, *p* = 0.0033; Figure 1A,B). No significant differences between oviposition periods were recorded when considering *T. japonicus* as host: both hyperparasitoid exploitation and hyperparasitism efficiency were low in the 1- and 3-day oviposition periods. Instead, when considering *T. mitsukurii* as host, *A. sinicus* displayed a mean hyperparasitoid exploitation efficiency of 46.1% in the 1-day period, and 96.3% in the 3-day period. The hyperparasitism efficiency of *A. sinicus* on *T. mitsukurii* was 25.9% in the 1-day oviposition period and 88.6% in the 3-day periods. *Trissolcus* emergence differed significantly between primary parasitoid species (χ^2^ = 1451.21, df = 1, *p* < 0.0001) and oviposition period (χ^2^ = 108.87, df = 1, *p* < 0.0001). The interaction between the two factors was also significant (χ^2^ = 55.61, df = 1, *p* < 0.0001; Figure 1C). Considering both 1- and 3-day exposure to *A. sinicus*, *T. japonicus* had an emergence rate of 94.4%, while for *T. mitsukurii* it was 53.9% and 3.7% in 1- and 3-day exposures, respectively.

**Figure 2:**
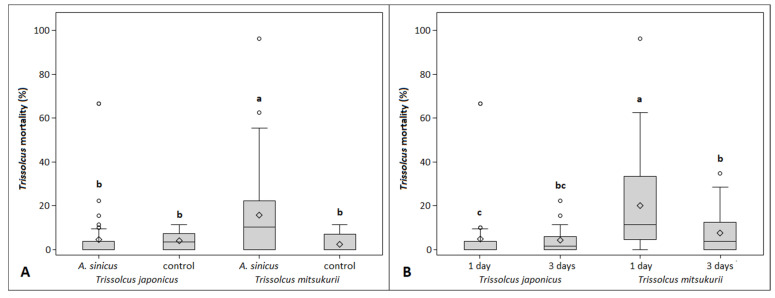
Boxplots showing *Trissolcus* mortality per egg masses exposed or not (“control”) to *A. sinicus*, observed during the first no-choice experiment for different *Trissolcus* species (**A**) and *A. sinicus* oviposition periods (**B**). Different letters indicate significant differences at Tukey–Kramer test on the least-square means (α = 0.05). Bars represent minimum and maximum scores. Circles indicate outliers.

The oviposition of *A. sinicus* also influenced developing primary parasitoids (χ^2^ = 13.02, df = 1, *p* = 0.0003), where the number of eggs with aborted *Trissolcus* was higher in egg masses that were exposed to the hyperparasitoids with respect to those that were not. Also, the interaction between hyperparasitism and *Trissolcus* species resulted significant on Trissolcus abortion rate (χ^2^ = 25.14, df = 1, *p* < 0.0001; Figure 2A). Natural mortality of *T. japonicus* was 4.1% per egg mass and 2.6% for *T. mitsukurii*. Egg masses exposed to *A. sinicus* registered 5.0% of aborted *T. japonicus* per egg mass and 15.8% of aborted *T. mitsukurii* per egg mass. Significant differences were observed also for the oviposition period effect (χ^2^ = 4.32, df = 1, *p* = 0.037) and the interaction between the oviposition period and *Trissolcus* species (χ^2^ = 16.32, df = 1, *p* < 0.0001; Figure 2B). The percentage of aborted *Trissolcus* was higher in the egg masses exposed to *A. sinicus* for the 1-day period as compared to the 3-day one, but this effect emerged only for *T. mitsukurii* (Figure 2B).

**Figure 3:**
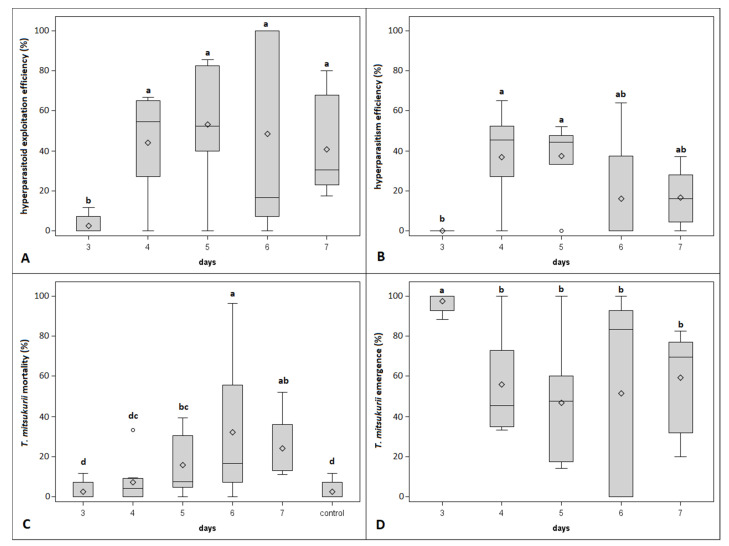
Boxplots showing the hyperparasitoid exploitation efficiency (**A**), hyperparasitism efficiency (**B**), mortality (**C**) and emergence (**D**) of *Trissolcus mitsukurii* observed on *T. mitsukurii*-parasitized egg masses at different days from primary oviposition during the second no-choice experiment (control represent the natural mortality of *T. mitsukurii*). Different letters indicate significant differences at Tukey–Kramer test on least-square means (α = 0.05). Bars represent minimum and maximum scores. Circles indicate outliers.

**Figure 4:**
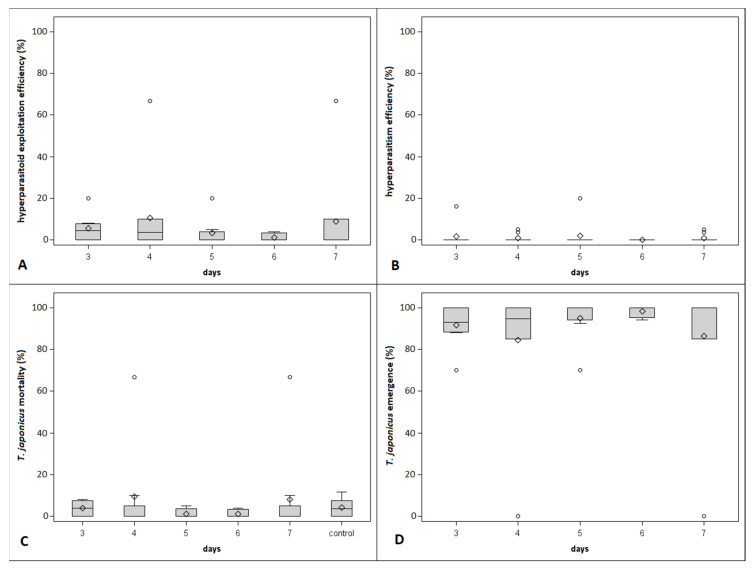
Boxplots showing the hyperparasitoid exploitation efficiency (**A**), hyperparasitism efficiency (**B**), mortality (**C**) and emergence (**D**) of *Trissolcus japonicus* observed on *T. japonicus*-parasitized egg masses at different days from primary oviposition during the second no-choice experiment (control represents the natural mortality of *T. japonicus*). Different letters indicate significant differences at Tukey–Kramer test on least-square means (α = 0.05). Bars represent minimum and maximum scores. Circles indicate outliers.

In the second no-choice test, 140 egg masses and 3233 eggs were inspected. *Acrocliosides sinicus* started to oviposit on *T. japonicus* on the 3rd day from the primary oviposition and lasted until the 7th day. In comparison, the hyperparasitization of *T. mitsukurii* started from the 4th day from the primary oviposition and lasted until the 7th day. No signs of hyperparasitism were observed in egg masses parasitized two days prior by either *T. japonicus* or *T. mitsukurii*, thus they were excluded from the analysis. As well as in the first no-choice test, hyperparasitism was superior on *T. mitsukurii* than on *T. japonicus* (hyperparasitoid exploitation efficiency: χ^2^ = 155.76, df = 1, *p* < 0.0001; hyperparasitism efficiency: χ^2^ = 38.03, df = 1, *p* < 0.0001; Figure 3 and Figure 4). Significant differences between *Trissolcus* species were observed also for the effect of the abortion with respect to natural abortion (i.e., control) and emergence (*Trissolcus* mortality: χ^2^ = 43.37, df = 1, *p* < 0.0001; *Trissolcus* emergence: χ^2^ = 155.76, df = 4, *p* < 0.0001; Figure 3 and Figure 4). All considered variables displayed significant differences between days from primary oviposition (hyperparasitoid exploitation efficiency: χ^2^ = 37.18, df = 4, *p* < 0.0001; hyperparasitism efficiency: χ^2^ = 11.20, df = 4, *p* = 0.0244; *Trissolcus* mortality: χ^2^ = 19.08, df = 5, *p* = 0.0019; *Trissolcus* emergence: χ^2^ = 37.18, df = 4, *p* < 0.0001; Figure 3 and Figure 4), but this was limited to egg masses previously parasitized by *T. mitsukurii* as shown by the significant interaction between the two factors (hyperparasitoid exploitation efficiency: χ^2^ = 66.00, df = 4, *p* < 0.0001; hyperparasitism efficiency: χ^2^ = 14.99, df = 4, *p* = 0.0047; *Trissolcus* mortality: χ^2^ = 51.10, df = 5, *p* < 0.0001; *Trissolcus* emergence: χ^2^ = 66.00, df = 4, *p* < 0.0001; Figure 3 and Figure 4).

### 3.2. Two-Choice Experiment

Most *A. sinicus* females (93%) used in this experiment chose an egg mass within 15 min from release in the arena, while only in few cases the choice was made after nearly one hour. Two replicates were not considered in the analysis because one of the two egg masses turned out to be not parasitized. Consistently with what was observed in the no-choice trial, the mean sex ratio of offspring in the two-choice trial was 88:12 female:male from *T. mitsukurii*-parasitized egg masses. The sex ratio of *A. sinicus* from *T. japonicus*-parasitized egg masses was 0:100 female:male.

Among the two host species, 97% of *A. sinicus* chose to oviposit on egg masses previously parasitized by *T. mitsukurii* (χ^2^ = 68.21, df = 1, *p* < 0.0001) and the remainder oviposited on *T. japonicus*. Furthermore, in egg masses previously parasitized by *T. japonicus*, the hyperparasitism efficiency by *A. sinicus* was 2.2%, while it was 34.2% in those parasitized by *T. mitsukurii*.

### 3.3. Olfactometer Trial

**Figure 5:**
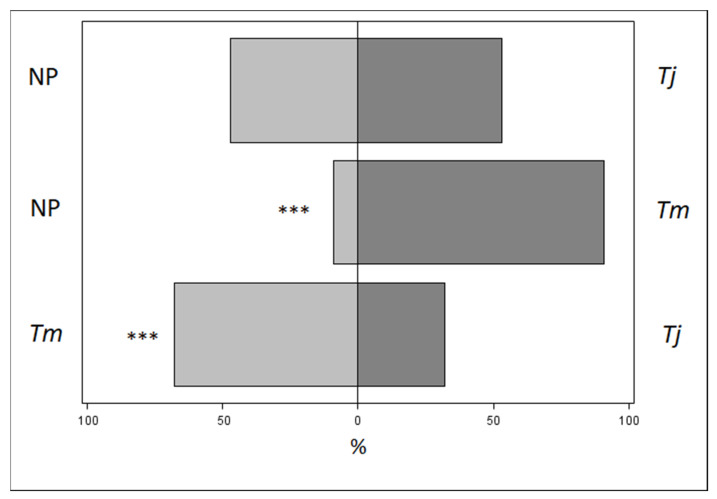
Response of *Acroclisoides sinicus* between the three different comparisons in olfactometer experiments. NP stands for not parasitized egg mass, *Tj* stands for *Trissolcus japonicus*-parasitized egg mass and *Tm* stands for *Trissolcus mitsukurii*-parasitized egg mass. Asterisks indicate significant differences at Fisher’s exact test (α = 0.05).

In the olfactometer, *A. sinicus* females exhibited a preference for egg masses parasitized by *T. mitsukurii* compared to those not parasitized (χ^2^ = 23.25, df = 1, *p* < 0.0001; Figure 5). No significant differences were observed between egg masses parasitized by *T. japonicus* and those not parasitized (χ^2^ = 0.18, df = 1, *p* = 0.6729; Figure 5). Comparing egg masses parasitized by *T. mitsukurii* with those parasitized by *T. japonicus, A. sinicus* showed a preference toward *T. mitsukurii* (χ^2^ = 12.20, df = 1, *p* = 0.0005; Figure 5).

### 3.4. Assessment of Acroclisoides sinicus Egg Load

**Figure 6:**
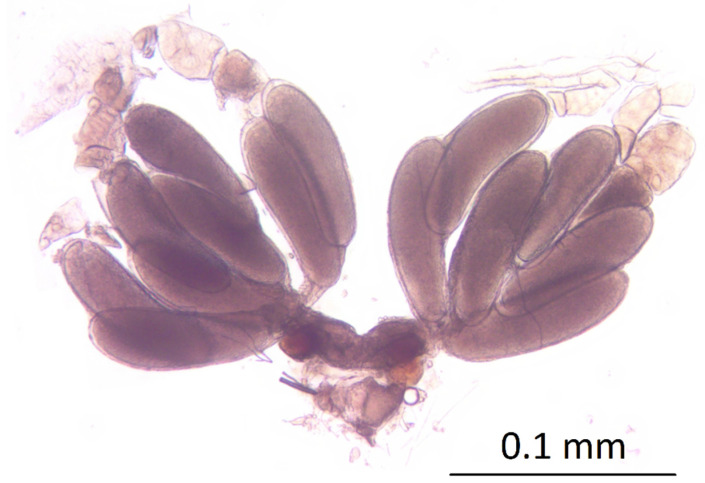
Ovaries of a 16-day-old *Acroclisoides sinicus* female, with visible mature eggs, part of ovarioles, oviducts and common oviduct.

**Figure 7:**
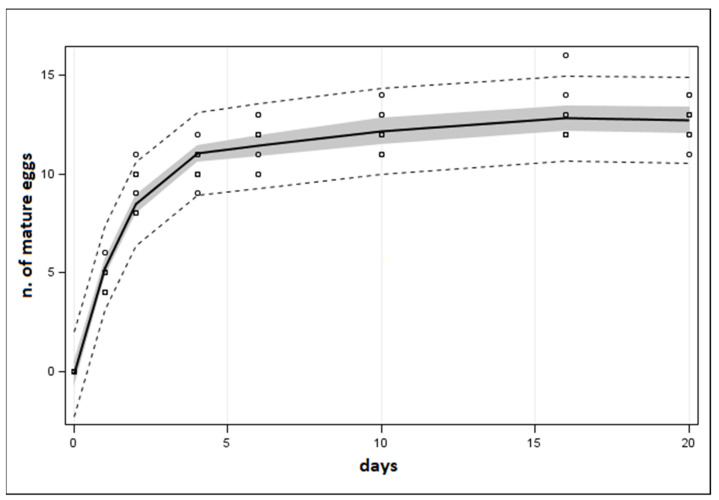
Polynomial regression ($y=0.0001x^{5}-0.0077x^{4}+0.1583x^{3}-1.5044x^{2}+6.7273x-0.1388$) of mature egg development in ovaries of Acroclisoides sinicus dissected females at different periods after the emergence from the host. During the experiment, females were never exposed to potential oviposition sites. The grey area represents the 95% confidence limit, broken lines represent the 95% prediction limit.

No mature eggs were found by dissecting *A. sinicus* females less than 24 h since emergence. However, one day after the emergence, 4.6 ± 0.2 S.E. mature eggs were found per female, and two days after the emergence, the number of mature eggs was 9.3 ± 0.3 S.E. Starting from the 4th day after the emergence, the mean number of mature eggs ranged from 10.4 ± 0.3 S.E. (post-emergence day 4) to 12.7 ± 0.3 S.E. (day 20; Figure 6). Mature egg load was highest in adults 16 days after the emergence, when they reached a maximum of 16 mature eggs per female (Figure 7).

The fitted regression was a 5th-order polynomial regression (F_1,5_ = 270.02, *p* < 0.0001; Figure 7) with R^2^ = 0.95. The egg load increased from the first to the 4th day and no further increases were observed between the 6th and 20th day.

## 4. Discussion

In this study, *A. sinicus* was confirmed as an obligate secondary parasitoid of *H. halys*. Its ability to develop on previously parasitized *H. halys* eggs demonstrates its potential negative influence on the success of biological control program against the brown marmorated stink bug [30,31]. In Europe, native egg parasitoids such as *A. bifasciatus* cannot provide satisfactory control of *H. halys*, even following augmentation programs [15,17,42]. The two Asian parasitoids, *T. mitsukurii* and *T. japonicus,* show higher performances and are currently considered for biological control programs that promise to provide higher control of *H. halys* [12,14,32]. Although the results of no-choice and two-choice trials identified both *T. mitsukurii* and *T. japonicus* as hosts of *A. sinicus*, the difference in developmental success between the two host species was remarkable. Furthermore, results of the two-choice experiment identified *T. mitsukurii* as the preferred host, with 97% of the *A. sinicus* tested females having laid their eggs into an egg mass previously parasitized by *T. mitsukurii* as compared to *T. japonicus*. *Acroclisoides sinus* was associated with various pentatomids other than *H. halys*. Thus, we cannot exclude the possibility that *A. sinicus* is able to act as a secondary or primary parasitoid on other hosts [25].

In no-choice trials, both hyperparasitoid exploitation and hyperparasitism efficiency significantly differed between the two host species. *Acroclisoides sinicus* showed a level of almost 25% of hyperparasitism efficiency on *T. mitsukurii* in the 1-day exposure, and it reached 89% with 3 days of oviposition. On the other hand, when considering *T. japonicus* as a host, both hyperparasitoid exploitation and hyperparasitism efficiency were close to zero. However, both *Trissolcus* species were able to emerge even in the case of high *A. sinicus* exploitation efficiency (i.e., 3-day oviposition trial on *T. mitsukurii*), and differences between the two species were detected. The emergence rate of *T. japonicus* was the same from both control and *A. sinicus*-exposed eggs. On the other hand, the hyperparasitoid almost eliminated the emergence of *T. mitsukurii* offspring in 1-day and 3-day exposure.

*Acroclisoides sinicus* increased the abortion of *T. mitsukurii* in cases with no emergence of adults. This could be related to direct host feeding, oviposition probing, venom injection, or laying eggs that fail to develop, potentially reducing the host survival [43,44,45,46,47]. However, it is difficult to determine the mechanisms of non-reproductive parasitoid-induced host mortality [40]. In this study, when *A. sinicus* emerged from the egg mass, *T. mitsukurii* experienced an average of 20% of abortion per egg mass. In contrast, aborted *T. japonicus* when exposed to *A. sinicus* were much less frequent (almost 5%) and this rate did not differ from the natural mortality of both *T. japonicus* and *T. mitsukurii*.

The results obtained in the second no-choice test confirm that *A. sinicus* is a hyperparasitoid of the pupal stage of *Trissolcus* sp. The differences among oviposition days can be explained by the host’s different developmental stages or ages [29]. No sign of hyperparasitism was observed in *H. halys* eggs that were parasitized by the primary parasitoid 2 days previously, since the *Trissolcus* pupae were probably not formed. It seems that the 4th and 5th day from primary oviposition is the optimal period for oviposition of *A. sinicus*, since high levels of hyperparasitism efficiency were observed. This could be related to the fact that 4 days are needed for the development of *Trissolcus* pupae [29]. Additionally, data from the second no-choice experiment suggest that 6th and 7th day-old pupae are not optimal for *A. sinicus* development.

The interpretation of results of host specificity trials for parasitoid wasps has been widely debated in literature [48,49,50]. However, no-choice experiments are considered the most informative and conservative, because even if they sometimes overestimate host ranges, they assess physiologically suitable hosts. Instead, results of choice experiments are generally considered biased because hosts’ cues may influence each other and thus the parasitoid behavior [51]. Many parasitoid wasps accept or reject an encountered host based on previous experience or habituation [50,52]. Nevertheless, several authors agreed that conducting both no-choice and two-choice tests provides an accurate estimate of the potential impact of a parasitoid on its hosts [48,50]. In the present study, *A. sinicus* revealed a strong preference for *T. mitsukurii*, in both no-choice and two-choice tests, with a potentially high impact on its development.

The olfactory response of *A. sinicus* females to different host sources displayed their capacity to distinguish between *T. mitsukurii* parasitized egg masses and an unparasitized egg mass. Moreover, when exposed simultaneously to volatiles emitted from *T. japonicus*, and unparasitized eggs, *A. sinicus* did not show a preference. Comparing egg masses parasitized by *T. japonicus* and *T. mitsukurii*, females of *A. sinicus* exhibited a preference in choosing *T. mitsukurii* volatiles. Hyperparasitoids can follow chemical cues in host location and can localize potential hosts [53,54]. The results obtained here showed that *A. sinicus* is able to use volatiles to distinguish egg masses parasitized by *T. mitsukurii* from those parasitized by *T. japonicus* or not parasitized. This could be related to marking substances emitted by *Trissolcus* species after oviposition [55]. Further research is needed to identify the mechanisms regulating host location in *A. sinicus*.

Ovary dissections showed that *A. sinicus* females within one day of emergence did not have mature eggs in their ovaries; thus, it should be considered as a synovigenic species. All species belonging to the family Pteromalidae considered by Jervis et al. [56] are synovigenic, and they usually store a limited number of mature eggs per ovariole for a limited period, since eggs often have a brief life. When a female is deprived of a host for an extended period, eggs were resorbed to make space for other mature oocytes. Based on these biological features, mature eggs observed 16 and 20 days after the emergence were probably of different ages, both fresh and old. Nevertheless, it should be noted that the number of observed eggs in each period reflects the effective maximum egg load of an *A. sinicus* female. Egg production of *A. sinicus* females may be stimulated by the presence of the male and the presence of the empty natal egg mass in which traces of the host are present [37], and this could explain the fact that the number of eggs was higher here than compared to Giovannini et al. [29], where *A. sinicus* egg load was studied on isolated females.

As previously stated, in *T. mitsukurii* parasitisation by *A. sinicus*, the 1-day oviposition period resulted in a lower hyperparasitism efficiency than the 3-day oviposition: the maximum number of eggs laid was 16 and 27, in 1 and 3 days, respectively. From the study of mature ovaries, an average of 12.7 ± 0.3 eggs (maximum 16) eggs per female were observed. Consequently, in 24 h of oviposition *A. sinicus* was able to lay all the eggs stored in the ovaries, and it seems unable to replenish ovaries for further oviposition. Instead, in the 3-day oviposition *A. sinicus* appears to lay all mature eggs and replenish its ovaries to almost entirely exploit the host egg mass. The rate of aborted *T. mitsukurii* was higher with 1-day exposure to *A. sinicus* compared to 3 days of exposure. We assume that high host availability stimulates stinging and venom injection of *A. sinicus* on more eggs than it can oviposit into [57]. These results may explain the *A. sinicus* behavior observed in the field: several hyperparasitoids females were found standing on *H. halys* egg masses without ovipositing (A.M. and D.S., personal observations). Females may wait on the egg mass for egg “replenishment” to fully exploit the egg mass parasitized by its host.

## 5. Conclusions

Biological control of *H. halys* with egg parasitoids is currently considered the most promising long-term solution against this pest. Among egg parasitoid species, *T. japonicus* and *T. mitsukurii* are the most effective biological control agents of *H. halys.* In this study, the hyperparasitoid *A. sinicus* displayed a clear preference for parasitizing *T. mitsukurii* compared to *T. japonicus*. Moreover, the hyperparasitism of *A. sinicus* negatively influences the emergence of *T. mitsukurii* but not that of *T. japonicus*. In a situation where both primary parasitoids of *H. halys* and this pteromalid are present, the hyperparasitoid may be expected to profoundly impact the composition of the parasitoid complex of *H. halys* with potential consequences on the outcome of biological control programs. Nevertheless, further study is needed to understand the potential impact of *A. sinicus* under field conditions.

## Data Availability

The data that support the findings of this study are available from the corresponding authors upon reasonable request.

## References

[1] Leskey T.C., Nielsen A.L. (2018). Impact of the invasive Brown Marmorated Stink Bug in North America and Europe: History, Biology, Ecology, and Management. Annu. Rev. Entomol..

[2] Maistrello L., Dioli P., Vaccari G., Caruso S. (2014). First records in Italy of the Asian Stinkbug *Halyomorpha halys*, a new Threat for Fruit Crops. ATTI Giornate Fitopatol..

[3] Maistrello L., Vaccari G., Caruso S., Costi E., Bortolini S., Macavei L., Foca G., Ulrici A., Bortolotti P.P., Nannini R. (2017). Monitoring of the invasive *Halyomorpha halys*, a new key pest of fruit orchards in northern Italy. J. Pest Sci..

[4] Bariselli M., Bugiani R., Maistrello L. (2016). Distribution and damage caused by *Halyomorpha halys* in Italy. EPPO Bull..

[5] Moore L., Tirello P., Scaccini D., Toews M.D., Duso C., Pozzebon A. (2019). Characterizing damage potential of the brown marmorated stink bug in cherry orchards in Italy. Entomol. Gen..

[6] Bosco L., Moraglio S.T., Tavella L. (2018). *Halyomorpha halys*, a serious threat for hazelnut in newly invaded areas. J. Pest Sci..

[7] Zapponi L., Tortorici F., Anfora G., Bardella S., Bariselli M., Benvenuto L., Bernardinelli I., Butturini A., Caruso S., Colla R. (2021). Assessing the Distribution of Exotic Egg Parasitoids of *Halyomorpha halys* in Europe with a Large-Scale Monitoring Program. Insects.

[8] Moraglio S.T., Tortorici F., Pansa M.G., Castelli G., Pontini M., Scovero S., Visentin S., Tavella L. (2020). A 3-year survey on parasitism of *Halyomorpha halys* by egg parasitoids in northern Italy. J. Pest Sci..

[9] Scaccini D., Falagiarda M., Tortorici F., Martinez-Sañudo I., Tirello P., Reyes-Domínguez Y., Gallmetzer A., Tavella L., Zandigiacomo P., Duso C. (2020). An insight into the role of *Trissolcus mitsukurii* as biological control agent of *Halyomorpha halys* in Northeastern Italy. Insects.

[10] Zhang J., Zhang F., Gariepy T., Mason P., Gillespie D., Talamas E., Haye T. (2017). Seasonal parasitism and host specificity of *Trissolcus japonicus* in northern China. J. Pest Sci..

[11] Avila G.A., Chen J.-H., Li W., Alavi M., Mi Q., Sandanayaka M., Zhang F., Zhang J. (2021). Seasonal Abundance and Diversity of Egg Parasitoids of *Halyomorpha halys* in Kiwifruit Orchards in China. Insects.

[12] Yang Z.Q., Yao Y.X., Qiu L.F., Li Z.X. (2009). A new species of *Trissolcus* (Hymenoptera: Scelionidae) parasitizing eggs of *Halyomorpha halys* (Heteroptera: Pentatomidae) in China with comments on its biology. Ann. Entomol. Soc. Am..

[13] Rice K.B., Bergh C.J., Bergmann E.J., Biddinger D.J., Dieckhoff C., Dively G., Fraser H., Gariepy T., Hamilton G., Haye T. (2014). Biology, ecology, and management of brown marmorated stink bug (Hemiptera: Pentatomidae). J. Integr. Pest Manag..

[14] Lee D.-H., Short B.D., Joseph S.V., Bergh J.C., Leskey T.C. (2013). Review of the Biology, Ecology, and Management of *Halyomorpha halys* (Hemiptera: Pentatomidae) in China, Japan, and the Republic of Korea. Environ. Entomol..

[15] Haye T., Fischer S., Zhang J., Gariepy T. (2015). Can native egg parasitoids adopt the invasive brown marmorated stink bug, *Halyomorpha halys* (Heteroptera: Pentatomidae), in Europe?. J. Pest Sci..

[16] Stahl J.M., Babendreier D., Haye T. (2018). Using the egg parasitoid *Anastatus bifasciatus* against the invasive brown marmorated stink bug in Europe: Can non-target effects be ruled out?. J. Pest Sci..

[17] Stahl J.M., Babendreier D., Marazzi C., Caruso S., Costi E., Maistrello L., Haye T. (2019). Can *Anastatus bifasciatus* be used for augmentative biological control of the brown marmorated stink bug in fruit orchards?. Insects.

[18] Moraglio S.T., Tortorici F., Visentin S., Pansa M.G., Tavella L. (2021). *Trissolcus kozlovi* in North Italy: Host Specificity and Augmentative Releases against *Halyomorpha halys* in Hazelnut Orchards. Insects.

[19] Talamas E.J., Herlihy M.V., Dieckhoff C., Hoelmer K.A., Buffington M., Bon M.-C., Weber D.C. (2015). *Trissolcus japonicus* (Ashmead) (Hymenoptera, Scelionidae) emerges in North America. J. Hymenopt. Res..

[20] Stahl J., Tortorici F., Pontini M., Bon M.-C., Hoelmer K., Marazzi C., Tavella L., Haye T. (2019). First discovery of adventive populations of *Trissolcus japonicus* in Europe. J. Pest Sci..

[21] Sabbatini Peverieri G., Talamas E., Bon M.C., Marianelli L., Bernardinelli I., Malossini G., Benvenuto L., Roversi P.F., Hoelmer K. (2018). Two Asian egg parasitoids of *Halyomorpha halys* (Stål) (Hemiptera, Pentatomidae) emerge in northern Italy: *Trissolcus mitsukurii* (Ashmead) and *Trissolcus japonicus* (Ashmead) (Hymenoptera, Scelionidae). J. Hymenopt. Res..

[22] Dieckhoff C., Wenz S., Renninger M., Reißig A., Rauleder H., Zebitz C.P.W., Reetz J., Zimmermann O. (2021). Add Germany to the List—Adventive Population of *Trissolcus japonicus* (Ashmead) (Hymenoptera: Scelionidae) Emerges in Germany. Insects.

[23] (2020). Decreto 42967 del 9 giugno 2020. Immissione in natura della specie non autoctona *Trissolcus japonicus* quale Agente di Controllo Biologico del fitofago *Halyomorpha halys* ai sensi del Decreto del Presidente della Repubblica 8 settembre 1997, n. 357, art. 12. http://agricoltura.regione.campania.it/difesa/files/DM_halyomorpha.pdf.

[24] Grissell E.E., Smith D.R. (2006). First report of *Acroclisoides* Girault and Dodd (Hymenoptera: Pteromalidae) in the western hemisphere, with description of a new species. Proc. Entomol. Soc. Washingt..

[25] Sabbatini Peverieri G., Mitroiu M.-D., Bon M.-C., Balusu R., Benvenuto L., Bernardinelli I., Fadamiro H., Falagiarda M., Fusu L., Grove E. (2019). Surveys of stink bug egg parasitism in Asia, Europe and North America, morphological taxonomy, and molecular analysis reveal the Holarctic distribution of *Acroclisoides sinicus* (Huang & Liao) (Hymenoptera, Pteromalidae). J. Hymenopt. Res..

[26] Clarke A.R., Seymour J.E. (1992). Two species of *Acroclisoides* Girault and Dodd (Hymenoptera: Pteromalidae) parasitic on *Trissolcus basalis* (Wollaston) (Hymenoptera: Scelionidae), a parasitoid of *Nezara viridula* (L.) (Hemiptera: Pentatomidae). Aust. J. Entomol..

[27] Tillman G., Toews M., Blaauw B., Sial A., Cottrell T., Talamas E., Buntin D., Joseph S., Balusu R., Fadamiro H. (2020). Parasitism and predation of sentinel eggs of the invasive brown marmorated stink bug, *Halyomorpha halys* (Stål) (Hemiptera: Pentatomidae), in the southeastern US. Biol. Control.

[28] Gariepy T.D., Haye T., Zhang J. (2014). A molecular diagnostic tool for the preliminary assessment of host-parasitoid associations in biological control programmes for a new invasive pest. Mol. Ecol..

[29] Giovannini L., Sabbatini-Peverieri G., Tillman P.G., Hoelmer K.A., Roversi P.F. (2021). Reproductive and Developmental Biology of *Acroclisoides sinicus*, a Hyperparasitoid of Scelionid Parasitoids. Biology.

[30] Sullivan D.J. (1987). Insect Hyperparasitism. Annu. Rev. Entomol..

[31] Nofemela R.S. (2013). The effect of obligate hyperparasitoids on biological control: Differential vulnerability of primary parasitoids to hyperparasitism can mitigate trophic cascades. Biol. Control.

[32] Sabbatini Peverieri G., Dieckhoff C., Giovannini L., Marianelli L., Roversi P.F., Hoelmer K. (2020). Rearing *Trissolcus japonicus* and *Trissolcus mitsukurii* for biological control of *Halyomorpha halys*. Insects.

[33] Talamas E.J., Buffington M.L., Hoelmer K. (2017). Revision of palearctic *Trissolcus* Ashmead (Hymenoptera, Scelionidae). J. Hymenopt. Res..

[34] Sabbatini Peverieri G., Giovannini L., Benvenuti C., Madonni L., Hoelmer K., Roversi P.F. (2020). Characteristics of the meconia of European egg parasitoids of *Halyomorpha halys*. J. Hymenopt. Res..

[35] Mayer C.J., Vilcinskas A., Gross J. (2008). Pathogen-induced Release of Plant Allomone Manipulates Vector Insect Behavior. J. Chem. Ecol..

[36] Belda C., Riudavets J. (2010). Attraction of the parasitoid *Anisopteromalus calandrae* (Howard) (Hymenoptera: Pteromalidae) to odors from grain and stored product pests in a Y-tube olfactometer. Biol. Control.

[37] Papaj D.R. (2000). Ovarian Dynamics and Host Use. Annu. Rev. Entomol..

[38] Jervis M.A., Copland M.J.W., Harvey J.A. (2007). The Life-cycle. Insects as Natural Enemies.

[39] Donaldson J.S., Walter G.H. (1988). Effects of egg availability and egg maturity on the ovipositional activity of the parasitic wasp, *Coccophagus atratus*. Physiol. Entomol..

[40] Bin F., Vinson S.B., Wajnberg E., Vinson S.B. (1991). Efficacy assessment in egg parasitoids (Hymenoptera): Proposal for a unified terminology. Proceedings of the Trichogramma and Other Egg Parasitoids 3rd International Symposium on Le Colloques de l’INRA.

[41] SAS Institute Inc. (2016). PROC User’s Manual.

[42] Costi E., Haye T., Maistrello L. (2019). Surveying native egg parasitoids and predators of the invasive *Halyomorpha halys* in Northern Italy. J. Appl. Entomol..

[43] Campbell R.W. (1963). Some Ichneumonid-Sarcophagid Interactions in the Gypsy Moth *Porthetria dispar* (L.) (Lepidoptera: Lymantriidae). Can. Entomol..

[44] Jervis M.A., Kidd N.A.C. (1986). Host-Feeding Strategies in Hymenopteran Parasitoids. Biol. Rev..

[45] Strand M.R., Ratner S., Vinson S.B. (1983). Maternally induced host regulation by the egg parasitoid *Telenomus heliothidis*. Physiol. Entomol..

[46] Asgari S., Rivers D.B. (2011). Venom Proteins from Endoparasitoid Wasps and Their Role in Host-Parasite Interactions. Annu. Rev. Entomol..

[47] Desneux N., Barta R.J., Hoelmer K.A., Hopper K.R., Heimpel G.E. (2009). Multifaceted determinants of host specificity in an aphid parasitoid. Oecologia.

[48] van Lenteren J.C., Bale J., Bigler F., Hokkanen H.M.T., Loomans A.J.M. (2006). Assessing risks of releasing exotic biological control agents of arthropod pests. Annu. Rev. Entomol..

[49] Louda S.M., Pemberton R.W., Johnson M.T., Follett P.A. (2003). Nontarget Effects—The Achilles’ Heel of Biological Control? Retrospective Analyses to Reduce Risk Associated with Biocontrol Introductions. Annu. Rev. Entomol..

[50] Murray T.J., Withers T.M., Mansfield S. (2010). Choice versus no-choice test interpretation and the role of biology and behavior in parasitoid host specificity tests. Biol. Control.

[51] Vinson S.B. (1976). Host Selection by Insect Parasitoids. Annu. Rev. Entomol..

[52] Abram P.K., Cusumano A., Abram K., Colazza S., Peri E. (2017). Testing the habituation assumption underlying models of parasitoid foraging behavior. PeerJ.

[53] Cusumano A., Harvey J.A., Bourne M.E., Poelman E.H., de Boer J.G. (2020). Exploiting chemical ecology to manage hyperparasitoids in biological control of arthropod pests. Pest Manag. Sci..

[54] Sullivan D.J., Völkl W. (1999). Hyperparasitism: Multitrophic ecology and behavior. Annu. Rev. Entomol..

[55] Rosi M.C., Isidoro N., Colazza S., Bin F. (2001). Source of the host marking pheromone in the egg parasitoid *Trissolcus basalis* (Hymenoptera: Scelionidae). J. Insect Physiol..

[56] Jervis M.A., Heimpel G.E., Ferns P.N., Harvey J.A., Kidd N.A.C. (2001). Life-history strategies in parasitoid wasps: A comparative analysis of “ovigeny”. J. Anim. Ecol..

[57] Liu W.-X., Wang W.-X., Zhang Y.-B., Wang W., Lu S.-L., Wan F.-H. (2015). Adult diet affects the life history and host-killing behavior of a host-feeding parasitoid. Biol. Control.

